# Annotated Chemical Patent Corpus: A Gold Standard for Text Mining

**DOI:** 10.1371/journal.pone.0107477

**Published:** 2014-09-30

**Authors:** Saber A. Akhondi, Alexander G. Klenner, Christian Tyrchan, Anil K. Manchala, Kiran Boppana, Daniel Lowe, Marc Zimmermann, Sarma A. R. P. Jagarlapudi, Roger Sayle, Jan A. Kors, Sorel Muresan

**Affiliations:** 1 Department of Medical Informatics, Erasmus University Medical Centre, Rotterdam, The Netherlands; 2 Fraunhofer-Institute for Algorithms and Scientific Computing (SCAI), Fraunhofer-Gesellschaft, Sankt Augustin, Germany; 3 RIA Medicinal Chemistry, AstraZeneca R&D Mölndal, Mölndal, Sweden; 4 GVK Biosciences Private Limited, Hyderabad, India; 5 NextMove Software Ltd, Cambridge, England; 6 Fraunhofer-Institute for Algorithms and Scientific Computing (SCAI), Fraunhofer-Gesellschaft, Sankt Augustin, Germany; 7 Chemistry Innovation Centre, AstraZeneca R&D Mölndal, Mölndal, Sweden; Macquarie University, Australia

## Abstract

Exploring the chemical and biological space covered by patent applications is crucial in early-stage medicinal chemistry activities. Patent analysis can provide understanding of compound prior art, novelty checking, validation of biological assays, and identification of new starting points for chemical exploration. Extracting chemical and biological entities from patents through manual extraction by expert curators can take substantial amount of time and resources. Text mining methods can help to ease this process. To validate the performance of such methods, a manually annotated patent corpus is essential. In this study we have produced a large gold standard chemical patent corpus. We developed annotation guidelines and selected 200 full patents from the World Intellectual Property Organization, United States Patent and Trademark Office, and European Patent Office. The patents were pre-annotated automatically and made available to four independent annotator groups each consisting of two to ten annotators. The annotators marked chemicals in different subclasses, diseases, targets, and modes of action. Spelling mistakes and spurious line break due to optical character recognition errors were also annotated. A subset of 47 patents was annotated by at least three annotator groups, from which harmonized annotations and inter-annotator agreement scores were derived. One group annotated the full set. The patent corpus includes 400,125 annotations for the full set and 36,537 annotations for the harmonized set. All patents and annotated entities are publicly available at www.biosemantics.org.

## Introduction

A substantial number of patent applications are filed every year by the pharmaceutical sector [1]. Exploring the chemical and biological space covered by these patents is crucial in early-stage medicinal chemistry activities [1], [2]. Patent specifications are one of many information sources needed to progress drug discovery projects. Patent analysis can provide understanding of compound prior art, novelty checking, validation of biological assays, and identification of new starting points for chemical exploration [3].

Extracting chemical and biological entities from patents is a complex task [4], [5]. Different approaches are currently used including manual extraction by expert curators, text mining supported by chemical and biological named entity recognition, or combinations thereof [6]. Chemical patents are complex legal documents that can contain up to hundreds of pages. The European Patent Office (EPO) [7], the pharmaceutically relevant patents within the United States Patent and Trademark Office (USPTO) [8], and the World Intellectual Property Organization (WIPO) [9] can be accessed and queried on-line via their websites. The patents are freely available from the patent offices, usually as XML, HTML or image PDFs, although EPO limits the number of downloads per week for non-paying users. Using optical character recognition (OCR), the image PDFs can be prepared for text mining. In fact, the available HTML and XML documents are mainly the OCR output prepared and published by the patent offices.

However, the text mining itself is a rather challenging task [10], [11]. Methods and their output can suffer dramatically from the large number of complex chemical names, term ambiguities, complex syntactic structures and OCR errors [12].

To validate the performance of named entity recognition techniques, the availability of a manually annotated patent corpus is essential [13]. Producing such annotated text is laborious and expensive. Most of the prior focus on corpora development has been on genes and proteins and less effort has been put into creating corpora for chemical terms [14]. Among the latter efforts, Kim et al. [15] in 2003 developed the GENIA corpus consisting of several classes of chemicals. The BioIE corpus by Kulick et al. [16] was made available in 2004 and included annotations of chemicals and proteins. In 2008, Kolárik et al. [17] released a small corpus of scientific abstracts annotated with chemical compounds. Recently, the CHEMDNER corpus, annotated with different classes of chemicals, was made available as part of the BioCreative challenge [18]. All these corpora consist of scientific abstracts from Medline. In a collaborative project between the EPO and the Chemical Entities of Biological Interest (ChEBI) in 2009 [19] a chemical patent corpus containing annotations of chemical entities and, if possible, their mapping to ChEBI chemical compounds [20] was developed. In a later study [21], the updated version of ChEBI [22] was used to increase the number of mappings. A larger patent corpus was developed in 2012 by Kiss et al. [12] which included name entity recognition of generic chemical compounds.

To our knowledge, the development of a gold standard patent corpus has not been systematically tackled before. Among the obvious reasons for this are the length and complexity of the patent text. In previous attempts only limited number of chemicals have been annotated and subclasses have not been defined. Other biological entities such as diseases or modes of actions have not been included and errors due to misspellings or OCR procedures have not been considered. Most previous studies on annotated corpora did not provide insights into inter-annotator agreement. This information would be valuable in assessing and comparing the performance of text mining applications.

Here we present a gold standard annotated corpus of 200 full patents for benchmarking text mining performance. The patent corpus includes annotation of chemicals with subclasses, diseases, targets and modes of action. Also spelling mistakes and spurious line break due to OCR errors are annotated within this corpus. The full-text patents and annotated entities are publicly available at www.biosemantics.org.

## Methods and Materials

### Corpus development strategy

The development of the gold standard patent corpus consisted of several phases. First, annotation guidelines were developed and a set of 200 diverse patents was chosen. The patents were pre-annotated automatically and made available to four independent annotator groups. The annotator groups could choose to consider or disregard the pre-annotations. Two patents were used to refine the annotation guidelines. The remaining patents were distributed between multiple annotator groups in a way that a subset of 47 patents was annotated by at least three groups, from which harmonized annotations were derived. Inter-annotator agreement scores between the annotator groups and against the harmonized set were computed. One annotator group annotated the complete set of patents.

### Patent corpus selection

The GVK BIO target class database [23] was used as a starting point for patent corpus selection. Patents from the EPO [7], USPTO [8], and WIPO [9] are available through this database, which includes relationships between documents, assays, chemical structures, assignees and protein targets, manually abstracted by expert curators [1]. Within the database, patents are binned based on different classes of protein families such as kinases or GPCRs [23].

All English language patents containing between 10 and 200 exemplified compounds, with a named primary target, were selected from the GVK BIO database. We made sure that all compounds had a molecular weight below 1000 to bias towards small-molecule patents. We did not specify limits on the time of the application. Overall 28,695 patents fulfilled the above criteria.

Chemical patents are known to include long sentences with complex syntactic structure [12]. Individual companies may have different ways of writing patents and we wanted to include diversity over assignees in the corpora. Therefore, if assignees had written multiple patents for one primary target, only one was randomly kept and the rest was disregarded.

Based on these selection criteria we were left with 8,016 patents grouped in 11 target classes. To make sure that a collection of well-known patents are included in the corpus, 50 drug patents from Sayle et al. [24] were added. Subsequently patents were randomly picked from each target group with a minimum of 10 patents per group. The diversity of the final selection is shown in Table 1. The final set consists of 121 USPTO, 66 WIPO, and 13 EPO patents, and contains over 11,500 pages and 4.2 million words.

**Table 1:** Target class distribution of the 8,066 patents from which the final set was drawn.

Target class	Number of patents	Final selection
GPCR	3,569	20
Protease	1,093	17
Kinase	1,046	12
Ion-Channel	433	14
Oxidoreductase	404	17
Hydrolase	364	15
NHR	349	15
Transporters	323	18
Other	218	11
Transferase	152	12
Phosphatase	65	17
Drugs from Sayle et al. [24]	50	32
Total	8,066	200

The patents were downloaded from the sources (EPO, USPTO, and WIPO) in XML format. Whenever multiple consecutive line breaks were encountered, they were replaced with a single line break. Images were also removed for all patents.

### Annotated entities

We annotated all compounds, diseases, protein targets, and modes of actions (MOA) mentioned in the patents. Compounds were assigned to a number of subclasses based on how they are generated: systematic identifiers and non-systematic identifiers [25]. The following systematic identifiers were annotated: IUPAC names [26], such as “ammonium phosphate” or “2-[2-(4-{2-[ethyl(2-fluorobenzyl)amino]-2-oxoethoxy}phenyl)ethoxy]benzoic acid”; SMILES notations [27], such as “n1c[nH]cc1”; and InChI strings [28], [29], such as “InChI = 1S/C2H6O/c1-2-3/h3H,2H2,1H3”. We also annotated the following non-systematic identifiers: trademarks, such as “Aspirin”, “Mesupron”, and “Arimidex”; abbreviations, such as “DCM”, “TBTU” and “DMAP”; CAS numbers [30], [31], such as “7732-18-5”; formulas, such as “MgSO4”; registry numbers, such as “ly256548”; and generic names, such as “iodotamoxifen”, “cycloalkylamines” and “racemate”. Any mention of diseases, such as “diabetes”, protein targets, such as “trypsin”, and MOAs, such as “antagonist”, were also annotated. OCR errors were also annotated in terms of spelling mistakes and spurious line breaks.

### Annotation guidelines

Initial annotation guidelines were developed based on previous work [14], [16]–[18]. Two patents (US5023269 [32] and US4659716 [33]) were randomly chosen from the patent corpus for training the annotators and fine-tuning the annotation guidelines. The following rules were defined:

1. When an entity is nested or has an overlap with another entity, annotate the entity that is more specific and informative. For example “5-HT1D” should not be annotated as target when it is embedded within the target annotation of “5-HT1D Serotonin Receptors”.
2. Annotate simple IUPAC names such as “water, “ammonia”, and “ethanol”.
3. Prefixes should be included within annotations, for example “1,4-” in “1,4-butanediol”.
4. Simple formulas such as “NaOH” and “(NH4)2SO4”, should be annotated as Formulas.
5. Counterions, such as “acetate”, “oxalate”, “propionate”, should be annotated as IUPAC names.
6. Generic structures such as “4-halo-phenol” or “xylene”, should be annotated as Generic names.
7. Polymers, e.g., “Polystyrene”, should be annotated as Generic names.
8. Trivial names, e.g., “Sildenafil”, should be annotated as IUPAC names.
9. Enumerations, like “hydrochloric” and “hydrobromic” in “include inorganic acids such as hydrochloric, hydrobromic”, should be annotated as IUPAC names.
10. Elements like “N”, “O”, and “C” should not be annotated.
11. Misspelled terms should be annotated as spelling mistakes (e.g., “hydrobroml:c”).
12. Annotations spanning over multiple lines because of spurious line breaks should be annotated as one term and be tagged with spurious line breaks.
13. Extra white space should be annotated as spelling mistakes (e.g., “hydro bromic”).
14. Do not annotate a term if it is splitted due to reasons other than OCR errors.
15. All symbols such as comma, charge symbol or brackets, should be included in the annotation (e.g., “n1c[nH]cc1”).

### Annotation process

Each patent was automatically pre-annotated using LeadMine (NextMove Software, UK) [34]. LeadMine can identify chemicals, protein targets, genes, species, company names, and also has the ability to recognise terms with spelling mistakes and suggest corrections. This increases the likelihood of detecting terms with OCR errors by the human annotator.

A pre-annotation consists of the span of text corresponding with the entity and its location within the text file. The following entity types were pre-annotated by LeadMine: IUPAC names, trivial names, CAS numbers, registry numbers, generic names, formulas, and targets. We did not pre-annotate SMILES and InChIs, as they are rarely present in patents. Diseases, and MOAs were also not included as this was not possible through our version of LeadMine.

**Figure 1:**
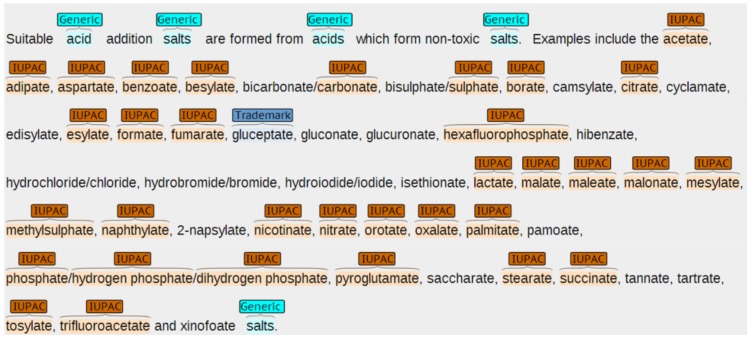
Example patent text with pre-annotations as shown by the Brat annotation tool.

For the annotation process the Brat rapid annotation tool (version 1.3) was used [35]. Brat allows online annotation of text using pre-defined entity types. It can display the pre-annotations and annotators can add new annotations and modify or delete the pre-annotated entities. To reduce mistakes and increase readability each entity type was marked by a specific color. For performance reasons we split the patents into pages with 50 paragraphs for display in Brat. Figure 1 shows a screenshot of Brat with pre-annotations.

Patents were annotated by annotators from four groups: AstraZeneca, Fraunhofer, GVK BIO, and NextMove. The GVK BIO annotation group consisted of ten annotators, while the other annotator groups had two annotators. One annotator group (Fraunhofer) chose to disregard the pre-annotations made by LeadMine. The patents were distributed between annotators within a group, such that each patent was annotated by only one annotator in a group. In the context of this paper, annotator group will refer to any individual annotator within the group.

Annotators had to correct any misidentified pre-annotation and had to add annotations that were missed in the pre-annotation step. Entities containing misspellings or spurious line breaks were separately annotated.

### Resolving misannotation of ambiguous terms

After the completion of the annotations by all groups, a group of annotators reviewed the results to reduce the number of ambiguous terms within the corpus. A term is defined as ambiguous if different groups annotated it with different entity types throughout the corpus.

A list of ambiguously annotated terms was compiled and annotators were asked to review the list only based on the different entity types assigned to each ambiguous term (i.e., the context of the terms was not provided). The annotators had to classify each term in one of three groups:

1- None of the entity types assigned to the term is applicable. All annotations of the term were removed from the corpus. For example, “nitrogen” was annotated as both IUPAC and Generic multiple times throughout the corpus. However, either entity type is incorrect since the term is an element. Therefore annotations of nitrogen are removed from the corpus.
2- One entity type is applicable. All occurrences of the term within the corpus were assigned to this entity type. For example, the term “DMF” was assigned 43 times as Trademark, 289 times as Abbreviation, and once as Formula. Regardless of the context of the text, DMF is an abbreviation and therefore the entity type of the term was changed to Abbreviation throughout the corpus.
3- More than one entity type is applicable. Only term annotations with an entity type that is not applicable, were removed throughout the corpus. For example, the term “5-ht” has been annotated 17 times as Abbreviation, 25 times as Generic, and 23 times as Target. Depending on the context of the text, the term can be either Target or Abbreviation but not Generic. Therefore all annotations of the term as Generic were removed from the corpus.

### Harmonization

To develop the gold standard corpus, the annotations of the 47 patents annotated by more than three groups were merged into a harmonized set. The centroid algorithm described by Lewin et al. [36] was used for this purpose.

Briefly, the algorithm tokenizes the annotations of different annotators at the character level and counts the number of agreeing annotators over pairs of adjacent annotation-internal characters [36]. Calculating votes over annotation-internal character pairs and not individual characters, guarantees that boundaries (starting and ending position of an annotated entity type) are considered in situations where two terms are annotated directly adjacent to each other [36]. The harmonized annotation consists of the characters pairs that have a vote equal to or larger than a specified threshold. In this work, we used a voting threshold of two, i.e., at least two annotators had to agree on the annotation.

The centroid algorithm was executed separately for each entity type. Therefore votes were only calculated if at least two annotators annotated a term with the same entity type.

### Inter-annotator agreement

Similar to Corbett et al. [14] and Kolárik et al. [17], we used the F-score (harmonic mean of recall and precision) to calculate the inter-annotator agreement between the annotator groups and between each annotator group and the harmonized set. For the comparison of two sets of annotations, one set was arbitrarily chosen as the gold standard (this choice does not affect the F-score). An annotation in the other set was counted as true positive if it was identical to the gold standard annotation, i.e., if both annotations had the same entity type and the same start and end location. If a gold standard annotation was not given, or not rendered exactly in the other set (i.e., non-matching boundaries or a different entity type), it was counted as false negative; if an annotation found in the other set did not exactly match the gold standard, it was counted as false positive.

## Results

### Patent distribution among groups

The number of annotated patents varied between annotation groups. Apart from the two patents used for training, 27 patents were annotated by NextMove, 36 by Fraunhofer, 49 by AstraZeneca, and 198 by GVK BIO. A total of 47 patents were annotated by at least three of the groups (three patents were annotated by all four groups).

### Initial harmonized set

The initial harmonized set, prior to disambiguation, was generated over the 47 common patents, yielding a total of 35,337 annotations (Table 2). The results show that IUPAC names and generic names have been annotated significantly more than any other chemical type, as has also been shown previously [13]. On the other hand, InChIs, CAS registry numbers and SMILES are rarely seen in these chemical patents. Also, a considerable number of diseases, targets, and MOAs have been annotated.

**Table 2:** Number of annotated terms and unique terms within the harmonized set prior to disambiguation.

Entity type	Annotated terms	Unique terms
IUPAC	14,423	5,365
Generic	7,959	880
Disease	3,777	1,257
Target	3,227	705
Trademark	2,273	987
Abbreviation	1,460	153
Formula	1,069	171
MOA	1,014	211
Registry Number	108	90
SMILES	21	21
CAS	6	5
InChI	0	0
Total	35,337	9,845

### Inter-annotator agreement prior to disambiguation

Table 3 shows the inter-annotator agreement between the groups and the harmonized set prior to disambiguation. There is generally a higher inter-annotator agreement between individual annotator groups and the harmonized set than between pairs of groups. The best agreement was 0.78. The agreement between groups ranged between 0.39 and 0.69. Investigation of the reasons for some low agreements suggested that adding a disambiguation step could resolve some of these disagreements.

**Table 3:** Inter-annotator agreement (F-score) without ambiguity resolution.

	AstraZeneca	Fraunhofer	GVK BIO		NextMove
Fraunhofer	0.42				
GVK BIO	0.60	0.39			
NextMove	0.50	0.69	0.52		
Harmonized	0.78	0.64	0.74	0.72	

### Disambiguation

A set of 2,135 unique ambiguous terms, corresponding to 47,044 annotations, were provided to annotators for disambiguation as described above. The annotators were able to make a decision for 333 unique ambiguous terms, affecting 9,005 annotations. The results in Table 4 show that most difficulties within the annotations were encountered between IUPAC names, Generic names and Trademarks. Also 23 elements were found that had been annotated 2,499 times with different entity types throughout the corpus. Since elements should not be annotated according to the guidelines, these terms were removed from the corpus.

**Table 4:** The effect of the disambiguation process on the annotations.

Rules	Type	Affected Terms	Affected Annotations
Add	IUPAC	52	2,275
annotation	Abbreviation	29	1,631
	Generic	67	976
	Trademark	71	442
	Disease	4	387
	MOA	2	203
	Formula	25	177
	Registry Number	28	111
	Target	19	32
Remove	Elements	23	2,499
annotation	IUPAC	7	103
	Trademark	3	101
	Generic	2	67
	Target	1	1
Total		333	9,005

### Inter-annotator agreement after disambiguation

After resolving the ambiguous terms, the harmonized set was recalculated. This resulted in an increase of inter-annotator agreement scores by 0.01 to 0.09 points (Table 5).

**Table 5:** Inter-annotator agreement after ambiguity resolution.

	AstraZeneca	Fraunhofer	GVK BIO		NextMove	Harmonized
AstraZeneca		+ 0.04	+ 0.09	+ 0.08		+ 0.06
Fraunhofer	0.46		+ 0.05	+ 0.03		+ 0.01
GVK BIO	0.69	0.44		+ 0.06		+ 0.05
NextMove	0.58	0.72	0.58			+ 0.03
Harmonized	0.84	0.65	0.79	0.75		

The lower left triangle presents the inter-annotator agreement scores (F-score). The upper right triangle shows the improvement gained through disambiguation.

Recalculating the inter-annotator agreement by only considering text boundaries and disregarding the entity types, further increases the agreement with up to 0.04 points. To analyze the reasons behind some of the low agreements, inter-annotator agreement scores were calculated for the main entity types (Table 6). The major difficulty in the annotation was encountered for non-systematic identifiers and MOAs, while identification of targets, diseases, and systematic identifiers were made with higher agreements.

**Table 6:** Inter-annotator agreement (F-score) between the harmonized set and the annotator groups for the main entity types.

	AstraZeneca Harmonized	Fraunhofer Harmonized	GVK BIO Harmonized	NextMove Harmonized
Overall	0.84	0.65	0.79	0.75
Chemicals	0.89	0.65	0.78	0.75
Systematic	0.94	0.81	0.91	0.93
Non-systematic	0.85	0.38	0.68	0.56
Disease	0.47	0.82	0.87	0.86
Targets	0.76	0.57	0.81	0.86
MOA	0.65	0.29	0.67	0.17

The inter-annotator agreement between the groups and overall, chemicals and systematic names were between 0.65 and 0.94. The inter-annotator agreement for non-systematic terms between Fraunhofer and the harmonized set was only 0.38. To investigate the reasons behind this low agreement, we recalculated the inter-annotator agreement between Fraunhofer and the harmonized set by considering cases where one annotation was embedded within the other annotation as an agreement. This only increased the inter-annotator score to 0.46. Further analysis showed that counting annotations that overlap as an agreement increased the score to 0.62. The main reason for the remaining differences was that annotators at Fraunhofer did not annotate formulas and had low agreements with others within the generic terms.

Table 6 shows that apart from AstraZeneca, all groups managed to gain a high inter-annotator agreement (0.82 to 0.86) between diseases and the harmonized set. Further analysis showed that the low inter-annotator agreement between AstraZeneca and the harmonized set on diseases is due to annotation differences in the boundaries. Calculating inter-annotator agreement on diseases by also accepting embedded terms increased the agreement to 0.70.

The inter-annotator agreement between Fraunhofer and the harmonized set for targets was only 0.57. Additional investigation showed that accepting embedded terms increased the agreement to 0.64.

The annotations of MOA for Fraunhofer and NextMove were also greatly affected by how the boundaries were chosen. An example is the term “mixed agonist” for which one group annotated the whole term as MOA and the other only annotated “agonist” as MOA. Accepting such cases as an agreement increases the agreement between NextMove and the harmonized set from 0.17 to 0.72, and between Fraunhofer and the harmonized set from 0.29 to 0.62.

### The gold standard patent corpus

The gold standard patent corpus consists of two sets: the harmonized corpus and the full corpus. The harmonized corpus consists of 47 patents with a total of 36,537 annotations for 9,813 unique terms (Table 7). In addition, 1,239 OCR errors have been annotated, of which 1,189 are spelling mistakes. The full patent corpus of 198 patents contains only the GVK BIO annotations with 400,125 annotations for 80,977 unique terms. The set includes 5,096 OCR error annotations, of which 4,403 are spelling mistakes.

**Table 7:** Number of annotated terms and unique terms in the harmonized set and in the full patent set of the gold standard corpus after disambiguation.

	Harmonized set (47 Patents)		Full set (198 Patents)	
	Unique terms	Annotated terms	Unique terms	Annotated terms
IUPAC	5,325	14,377	50,893	135,603
Generic	881	8,384	14,305	169,133
Disease	1,256	3,776	4,503	20,229
Target	703	3,235	3,514	14,398
Trademark	994	2,366	3,365	9,574
Abbreviation	153	2,088	778	21,087
Formula	169	1,127	3,108	25,716
MOA	210	1,017	110	3,837
Registry Number	96	140	188	329
SMILES	21	21	166	166
CAS	5	6	47	53
InChI	0	0	0	0
Total	9,813	36,537	80,977	400,125

## Discussion and Conclusions

We have produced a gold standard chemical patent corpus consisting of 198 full patents of which 47 patents have been annotated by at least three annotators. The patent corpus contains a selection of patents from WIPO, USPTO and EPO with annotation of compounds, diseases, targets, and MOAs. We have also annotated spelling errors for the mentioned entity types.

We have released the inter-annotator agreements along with the gold standard corpus. Making inter-annotator agreement scores available will hopefully prove to be useful for performance assessment of automatic annotations of the patent corpus.

To our knowledge this is the first patent gold standard corpus containing full patents with different entity types (chemicals and their sub entities, diseases, MOAs, and targets). Patents are one of the richest knowledge sources with high information content and detailed description of chemistry and technology. Our annotation process showed the complexity of the annotation task. The OCR process added a significant level of noise to the text. A high inter-annotator agreement was seen on the annotation of entities such as systematic names. In contrast, we observed lower inter-annotator agreements for non-systematic names and MOAs. This emphasizes the challenges in identifying named entities from patent text. Annotation of OCR errors may also be helpful to improve patent informatics systems by facilitating the development of algorithms to correct such errors.

The annotated gold standard corpus should prove a valuable resource for developing and evaluating patent text analytics approaches.

### Availability

The gold standard corpus is available through www.biosemantics.org.
